# Interaction of Ingested Leucine with Glycine on Insulin and Glucose Concentrations

**DOI:** 10.1155/2014/521941

**Published:** 2014-07-10

**Authors:** Jennifer F. Iverson, Mary C. Gannon, Frank Q. Nuttall

**Affiliations:** ^1^Endocrinology, Metabolism, and Nutrition Section, Minneapolis VA Health Care System, One Veterans Drive, Minneapolis, MN 55417, USA; ^2^Department of Medicine, University of Minnesota, Minneapolis, MN 55455, USA; ^3^Department of Food Science and Nutrition, University of Minnesota, St. Paul, MN 55108, USA

## Abstract

The majority of individual amino acids increase insulin and attenuate the plasma glucose response when ingested with glucose.* Objective.* To determine whether ingestion of two amino acids simultaneously, with glucose, would result in an additive effect. Leucine (Leu) and glycine (Gly) were chosen because they were two of the most potent glucose-lowering amino acids when given individually.* Materials and Methods.* Nine subjects received test items on four separate days. The first was a water control, then 25 g glucose, or Leu + Gly (1 mmol/kg fat-free mass each) ±25 g glucose, in random order. Glucose, insulin, and glucagon were measured frequently for 2.5 hours. Net areas were calculated.* Results.* The glucose area response decreased by 66%. The insulin area response increased by 24% after ingestion of Leu + Gly + glucose compared to ingestion of glucose alone. The decrease in glucose response was not additive; the increase in insulin response was far less than additive when compared to previously published individual amino acid results. The glucagon concentration remained unchanged.* Conclusion.* There is an interaction between Leu and Gly that results in a markedly attenuated glucose response. This occurred with a very modest increase in insulin response. Changes in glucagon response could not explain the results. The mechanism is unknown.

## 1. Introduction

In a series of studies our laboratory has demonstrated that single amino acids when ingested at 1.0 mmol/kg fat free mass with or without 25 g of glucose stimulate a rise in insulin and glucagon. When ingested with glucose they are capable of attenuating the glucose rise integrated over a 2.5-hour period of time. Sixteen amino acids were studied. The potency of the individual amino acids in regard to their effect on the insulin, glucagon, and glucose concentrations varied greatly between amino acids and the specific responses could not be predicted based on the structure of the amino acids [1].

Leucine is well recognized to strongly stimulate insulin secretion and to reduce the glucose rise when ingested with glucose [2]. When ingested with glucose, glycine similarly reduced the glucose rise, but only weakly stimulated insulin secretion. [2, 3]. Therefore, we were interested in determining if the glucose-attenuating effect would be greater if these two amino acids were ingested together with glucose. We were particularly interested in whether the effects would be additive since glycine produced a major decrease in glucose response in the presence of only a very modest increase in insulin; that is, it may reduce glucose by an insulin-independent mechanism. The effect of these amino acids on the insulin and glucagon responses also was monitored.

## 2. Methods

Nine healthy subjects (5 men and 4 women) were studied. The mean age was 23.2 years (range 18–30). Their mean weight was 71.9 kg (range 57.2–96.4) with a mean fat-free mass of 52.5 kg (range 40.9–66.8) and a mean body mass index of 23.5 (range 19.7–27.2).

Written informed consent was obtained from all subjects. The study was approved by the Minneapolis VA Health Care System and the University of Minnesota Committees on Human Subjects and filed in the ClinicalTrials.gov Registry (NCT01471509).

Fat-free mass was determined using a Bioelectrical Impedance Analyzer (RJL Systems, Inc., Clinton Township, MI, USA). Baseline laboratory studies including thyroid, liver, and kidney function as well as fasting plasma glucose, hemoglobin A1c, and lipid profiles were within the normal reference ranges.

Each subject was admitted to the Special Diagnostic and Treatment Unit (SDTU) on four separate mornings. Subjects were asked to fast for 12 hours prior to testing. On the first day subjects were given water only as a fasting control. At subsequent visits the following three test meals were given in random order: (1) 25 g glucose (45 mL Glutol), (2) L-leucine + glycine (1 mmol/kg fat-free mass each), and (3) 25 g glucose with L-leucine + glycine (1 mmol/kg fat-free mass each). All were ingested with a total of 120 mL of water. All amino acids were kindly provided by Ajinomoto, Inc. (Raleigh, NC, USA). The amino acid dose of 1 mmol/kg fat-free mass was chosen because it is approximately the amount of an amino acid that would be consumed during a high-protein meal. An indwelling catheter was inserted into an antecubital vein and kept patent with intravenous saline. Baseline blood samples were drawn at 7:40, 7:50, and 8:00 AM. Then, blood samples were obtained every ten minutes for 120 minutes and at 150 minutes after ingestion of each test meal for determination of plasma glucose, serum insulin, and plasma glucagon concentrations.

Net areas under the curve for each parameter were calculated using a computer program based on the trapezoid rule with the mean fasting value used as baseline. The area of the water control was subtracted from the area of the test substances to give the true area response. Data for each amino acid pair was then compared to data we had previously obtained for each of the component individual amino acids [2, 3]. Ideally, we wanted to study each subject for an additional four days to allow comparisons between the individual amino acids and the amino acid pairs in the same subjects rather than having to compare the data with historical data. Unfortunately, however, given the longer time commitment required and difficulty in recruiting subjects for an eight-day study, this design was not practical.

Plasma glucose concentrations were determined by a hexokinase method using an Abbott Architect ci8200 analyzer (Abbott Laboratories, Abbott Park, IL, USA). Serum immunoreactive insulin was measured using an automated chemiluminescent assay on Siemens' IMMULITE analyzer. Plasma glucagon was determined by radioimmunoassay using kits purchased from Millipore (Billerica, MA, USA).

Statistics were determined using Student's *t*-test for paired variates with the StatView 512+ program (Abacus Concepts, Calabasas, CA, USA) for the Macintosh computer (Apple, Cupertino, CA, USA). A *P* value less than 0.05 was the criterion used for significance. Data are presented as means ± SEMs.

## 3. Results

**Figure 1:**
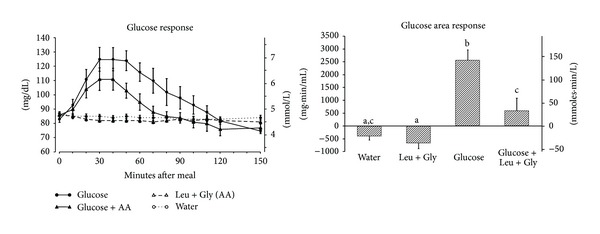
(left) Mean (± SEM) plasma glucose concentration in 9 healthy subjects after ingestion of water only (open circles), 25 g glucose (closed circles), leucine + glycine at 1 mmol/kg fat-free mass each (open triangles), or 25 g glucose with leucine + glycine at 1 mmol/kg fat-free mass each (closed triangles). (right) Net integrated AUC using the fasting values as baseline. Bars with different letters indicate values are significantly different (*P* < 0.05).

The mean fasting glucose concentration was 85 ± 0.9 mg/dL (Figure 1 left). After ingestion of glucose alone the mean plasma glucose concentration reached a maximum of 125 ± 8.3 mg/dL at 30–40 minutes and then slowly returned to the fasting concentration by 120 minutes. When the subjects ingested the same amount of glucose with leucine + glycine the maximum glucose concentration was reached at the same time, but was lower (111 ± 7.8 mg/dL). The plasma glucose concentration also returned to baseline more quickly (80 minutes versus 120 minutes) after ingestion of glucose with leucine + glycine compared to ingestion of glucose alone. Ingestion of water or leucine + glycine without glucose had little effect on plasma glucose levels.

The glucose area response (Figure 1 right) decreased by 66% when leucine + glycine were ingested with glucose compared to ingestion of glucose alone (*P* = 0.015). The glucose area response to leucine + glycine alone was slightly less than that for water alone, but this did not reach statistical significance.

**Figure 2:**
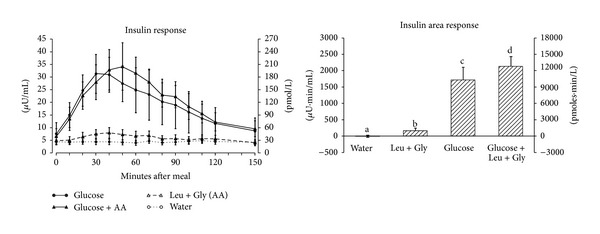
(left) Mean (± SEM) serum insulin concentration in 9 healthy subjects after ingestion of water only (open circles), 25 g glucose (closed circles), leucine + glycine at 1 mmol/kg fat-free mass each (open triangles), or 25 g glucose with leucine + glycine at 1 mmol/kg fat-free mass each (closed triangles). (right) Net integrated AUC using the fasting values as baseline. Bars with different letters indicate values are significantly different (*P* < 0.05).

The mean fasting serum insulin concentration was 5.9 ± 1.3 *μ*U/mL (Figure 2 left). After ingestion of glucose alone the mean serum insulin concentration reached a maximum of 31.3 ± 7.7 *μ*U/mL at 30 minutes then steadily decreased and was close to the baseline level by 150 minutes. When glucose was ingested with leucine + glycine the mean concentration reached a maximum of 34.2 ± 9.4 *μ*U/mL at 50 minutes after ingestion; it then rapidly declined and also was approaching the baseline concentration by 150 minutes. Ingestion of leucine + glycine without glucose resulted in a modest increase in serum insulin concentration to 8.0 ± 2.1 *μ*U/mL at 40 minutes after ingestion.

The insulin area response (Figure 2 right) increased by only 24% when leucine + glycine were ingested with glucose compared to glucose ingestion alone (*P* = 0.046). Ingestion of leucine + glycine without glucose resulted in a small but significantly higher insulin area response compared to water ingestion alone (*P* = 0.037).

**Figure 3:**
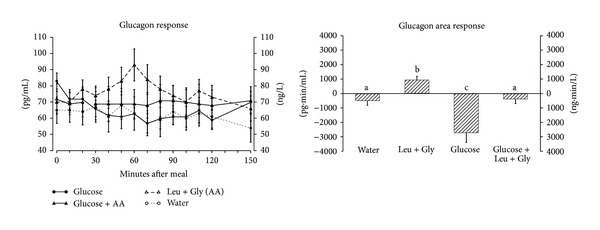
(left) Mean (± SEM) plasma glucagon concentration in 8 healthy subjects after ingestion of water only (open circles), 25 g glucose (closed circles), leucine + glycine at 1 mmol/kg fat-free mass each (open triangles) or 25 g glucose with leucine + glycine at 1 mmol/kg fat-free mass each (closed triangles). (right) Net integrated AUC using the fasting values as baseline. Bars with different letters indicate values are significantly different (*P* < 0.05).

The mean fasting glucagon concentration was 73 ± 3.7 pg/mL (Figure 3 left). After ingestion of glucose alone the plasma glucagon concentration decreased as expected and remained below the initial fasting value for the duration of the study. In contrast, ingestion of leucine + glycine alone resulted in an increase, but a return to baseline by the end of the 150 minute study period. The plasma glucagon concentration was attenuated when glucose was coingested with leucine + glycine and remained at a basal concentration.

The net glucagon area response (Figure 3 right) was more negative after ingestion of glucose alone compared to water (*P* = 0.018) and positive after ingestion of leucine + glycine alone (*P* = 0.003). When leucine + glycine were ingested with glucose the glucagon area response increased by 94% compared to ingestion of glucose alone (*P* = 0.016). It was not different from that when only water was ingested; that is, it neutralized completely the decrease observed after glucose ingestion. The glucagon area response after ingestion of leucine + glycine alone was slightly positive and significantly different from the water control (*P* = 0.003).

## 4. Discussion

### 4.1. Glucose Response

The coingestion of leucine + glycine with glucose decreased the glucose area response by 66%. In our previous studies we found that ingestion of leucine alone with glucose [2] or glycine alone with glucose [3] resulted in decreases in the glucose area response of 57% and 62%, respectively, when compared to ingestion of the same amount of glucose alone. Thus, the decrease in glucose area response with ingestion of leucine + glycine with glucose was essentially the same when compared to the amino acids ingested individually with glucose (leucine = 57%, glycine = 62%, leucine + glycine = 66%) (Table 1). More importantly, this occurred even though the insulin increase (24%) was less than that stimulated by leucine ingested independently with glucose (59%) in our previous study [2]. Thus, the smaller glucose area response must have been due to the glycine component.

The mechanism or mechanisms by which an ingested amino acid, such as glycine, can attenuate the glucose rise without an additional rise in insulin is an important issue, but is yet to be determined. In the present study it also attenuated the expected rise in insulin based on the data when leucine alone was ingested with glucose. It cannot be explained by any of the known gut hormones. They all stimulate an increase in insulin.

### 4.2. Insulin Response

Leucine, whether ingested [2] or given intravenously [4] has been demonstrated to stimulate insulin secretion and this is greatly facilitated by the simultaneous ingestion of glucose. Also a potential mechanism has been described [5–9]. Indeed, ingested leucine is the only amino acid known to directly stimulate insulin secretion [10].

The proposed mechanism is that leucine enters the beta cells and allosterically activates mitochondrial glutamate dehydrogenase (GDH). This results in an increased conversion of glutamate to *α* ketoglutarate and, thus, an increased flux of oxidative substrate into the mitochondria (anaplerosis). The resulting increased ATP production ultimately results in an increased release of insulin into the circulation [8, 10].

In contrast to leucine, the ability of glycine to increase insulin appears to be much more modest. Indeed, the ability of glycine to increase insulin has not been consistently demonstrated. In the present study and others [3, 11], oral administration of glycine did produce a modest increase in insulin while in 2 other studies glycine did not significantly affect the insulin concentration in healthy subjects [12, 13]. Also, intravenous administration of glycine to 2 overweight subjects did not stimulate an increase in insulin at a high physiologic concentration, but did modestly stimulate an increase in insulin at a supraphysiological concentration [14].

The possibility that stimulation of a gut-derived incretin hormone is involved in the mechanism by which oral glycine modestly increases insulin remains uncertain. However,* in vitro* data demonstrating that glycine can stimulate GLP-1 release from an intestinal L-cell model line GLUTag has been reported [15].

In our previous studies we determined that ingestion of 1 mmol/kg fat-free mass leucine with 25 g glucose resulted in an insulin area response that was 59% greater than that observed after the ingestion of the same amount of glucose alone (2), whereas, using the same protocol, ingestion of glycine with glucose had no additional effect on insulin area response (3). Thus, leucine enhanced the insulin response to glucose whereas glycine had no effect.

In the present study, the expected insulin response to leucine was markedly attenuated when compared to our previously obtained data (24% versus 59% increase in area response) (Table 1). Whether the effect by glycine is due to an insulin-independent lowering of the glucose response or a more direct inhibition of leucine-stimulated insulin secretion by the addition of glycine remains to be determined.

In this regard, a similar pattern was seen when the two amino acids were ingested without glucose. The insulin area response to leucine + glycine ingested together was 10% of that observed with glucose alone. In our previous studies the ingestion of leucine alone [2] or glycine alone [3] resulted in insulin area responses relative to glucose of 10% and 16%, respectively. Thus, the insulin area response following ingestion of leucine + glycine without glucose in the current study was less than additive (leucine = 10%, glycine = 16%, leucine + glycine = 10%) (Table 1). These data suggest, but do not prove, that there is a direct interaction between these two amino acids which results in an attenuated insulin response when ingested with glucose and this is not glucose-concentration dependent.

### 4.3. Glucagon Response

Overall, glucagon is considered to play a significant role in maintaining glucose homeostasis by serving as a counterbalance to insulin.

When proteins or individual amino acids are ingested the glucagon concentration rises, but the response varies considerably between proteins [16] and between amino acids [1]. When leucine [2] or glycine [3] was ingested individually we reported that each increased the glucagon concentration. In the present study when both were ingested, the glucagon response was the same as with leucine alone. The increase relative to the water control when the amino acids were ingested individually can be compared but cannot be quantified in an absolute sense since a different assay was used for the determination of the glucagon response to glycine. That assay resulted in higher values in all cases.

When only glucose is ingested, the glucagon concentration decreases. In general, the decrease resulting from glucose ingestion is attenuated when ingested with amino acids [1]. Of some interest, when leucine [2, 3] was ingested with glucose the net glucagon area response was not different from the area response to water. This also was the case in the present study when both amino acids, in the same amounts as used previously, were ingested together. Thus, there appears to be a mechanism present which maintains a constant, unchanged glucagon concentration regardless of the amino acid or its combination when ingested with glucose. It is possible that one amino acid is inhibiting the effect of the other. If so, it is carefully poised to balance the inhibiting effect of ingested glucose on the glucagon response.

## 5. Conclusion

Ingestion of leucine + glycine with glucose produced a similar attenuation in glucose response compared to when each amino acid was ingested alone with glucose. This could not be explained by a greater insulin response. Also the glucagon concentration remained unchanged rather than decreased, as when only glucose was ingested. Thus, neither an increased insulin response and/or a suppressed glucagon concentration can explain the results. Overall, the way in which ingested amino acids interact with each other and affect glucose metabolism is complex and is not well understood. It is at least partially insulin-independent.

## Figures and Tables

**Table 1:** Comparison of effects of single and two amino acids on glucose, insulin, and glucagon area responses.

Amino acid + glucose data						
	Glucose		Insulin		Glucagon	
	Area	%**↓**	Area	%**↑**	Area	%**↑**
Glycine publication∗						
Glucose	100%		100%		N/A^∧^	
Glucose + Gly	38%	62%	101%	1%		
Leucine publication∗∗						
Glucose	100%		100%		−100%	
Glucose + Leu	43%	57%	159%	59%	−7%	93%
Present publication∗∗∗						
Glucose	100%		100%		−100%	
Gluc + Leu + Gly	34%	66%	124%	24%	−6%	94%

Individual amino acid data						
	Glucose area		Insulin area		Glucagon area^∧∧^	

Glycine alone∗	−3%		16%		N/A^∧^	
Leucine alone∗∗	11%		10%		84%	
Leu + Gly∗∗∗	−9%		10%		67%	

*Gannon et al., 2002 [3].

**Kalogeropoulou et al., 2008 [2].

***Iverson et al., present paper.

The area response after ingestion of water only (control) was subtracted from the area response to the ingested test substances. All data are presented relative to the response to ingested glucose, which is set at +100% for glucose and insulin, and−100% for glucagon (because glucose ingestion results in a decrease in glucagon concentration).

^∧^N/A: not applicable: The glucagon data published in the glycine paper were obtained using an older assay and cannot be quantitatively compared to the data from the leucine paper or the present leucine + glycine paper.

^∧∧^Glucagon area when glucose alone is −100%.
